# Effects of the Severity of Stenosis on Clinical Outcomes of Indirect Decompression Using Oblique Lumbar Interbody Fusion

**DOI:** 10.3390/jcm13154421

**Published:** 2024-07-28

**Authors:** Dong-Ho Kang, Jonghyuk Baek, Bong-Soon Chang, Hyoungmin Kim, Seong Hwa Hong, Sam Yeol Chang

**Affiliations:** 1 Department of Orthopedic Surgery, Samsung Medical Center, Gangnam-gu, Seoul 06351, Republic of Korea; kang9451@snu.ac.kr; 2 Department of Orthopedic Surgery, Seoul National University College of Medicine, Jongno-gu, Seoul 03080, Republic of Korea; 3 Department of Orthopedic Surgery, Seoul National University Hospital, Jongno-gu, Seoul 03080, Republic of Korea

**Keywords:** oblique lumbar interbody fusion, indirect decompression, severe foraminal stenosis, severe central stenosis, substantial clinical benefit, foraminal osteophyte

## Abstract

**Background:** No consensus has been reached regarding the efficacy of indirect decompression through oblique lumbar interbody fusion (OLIF) in severe lumbar spinal stenosis (LSS). This study investigated the impact of preoperative magnetic resonance imaging (MRI)-based grading of central and foraminal stenosis on OLIF outcomes in LSS patients and identified risk factors for postoperative clinical dissatisfaction. **Methods:** We retrospectively reviewed LSS patients who underwent OLIF with a minimum 1-year follow-up. Clinical scores obtained preoperatively and at 3, 6, 12, and 24 months postoperatively were analyzed using the substantial clinical benefit (SCB) framework. The severity of central and foraminal stenosis in the initial MRI was assessed through qualitative grading systems. **Results:** Among the 145 patients, with a mean follow-up of 33.7 months, those with severe central stenosis showed a significantly higher proportion of patients achieving SCB in the visual analog scale for leg pain (94.5% versus 83.1%; *p* = 0.044) at one year postoperatively than those without. However, those with severe foraminal stenosis showed significantly higher Oswestry Disability Index (ODI) scores (*p* = 0.024), and lower walking ability scores in the Japanese Orthopedic Association Back Pain Evaluation Questionnaire (JOABPEQ) (*p* = 0.004) at one year postoperatively than those without. The presence of a foraminal osteophyte of the superior articular process (SAP) was a significant risk factor responsible for not achieving SCB in ODI and walking ability in JOABPEQ at one year postoperatively (odds ratio: 0.20 and 0.22, respectively). **Conclusions:** After OLIF, patients with severe central stenosis showed clinical outcomes comparable to those without. The improvement in ODI and walking ability in JOABPEQ was limited in patients with severe foraminal stenosis. Surgeons should consider direct decompression in cases with the presence of foraminal osteophytes of SAP.

## 1. Introduction

Lumbar spinal stenosis (LSS) is the most common disease associated with lower back pain, radiating pain in the lower extremities, and gait disturbance in elderly patients [1,2]. LSS results from the narrowing of the spinal canal, which compresses nerve roots and the spinal cord, causing significant discomfort and functional impairment. Its increasing incidence due to the aging population poses a significant healthcare challenge [3]. Lumbar interbody fusion is a safe and effective treatment option for some patients with LSS. Among the various lumbar interbody fusion surgeries, lateral access surgery, such as oblique lumbar interbody fusion (OLIF), is called “indirect decompression” because it does not involve the direct removal of the pathological structures compressing the spinal canal [4]. Indirect decompression is achieved by the restoration of the intervertebral disc (IVD) height and foraminal height [4,5,6], as well as gradual remodeling of the spinal canal following stabilization [7,8,9].

In previous literature, whether indirect decompression is sufficient is controversial, especially in cases of severe stenosis. Previous studies have reported that severe stenosis, osteophytes around the endplate and intervertebral foramen, and severe facet arthropathy are risk factors for additional posterior decompression following initial indirect decompression [10,11,12,13]. In contrast, more recent studies have reported that indirect decompression alone can also be effective, even in patients with severe spinal stenosis [14,15,16].

Buckland et al. reported that one of the pitfalls with indirect decompression is the presence of foraminal osteophytes from the posterior to the cephalad vertebral body causing nerve root compression [12]. Few studies have evaluated which anatomical factors in severe foraminal stenosis are associated with poor outcomes after indirect decompression [12,13]. Four main anatomical factors are responsible for foraminal stenosis in patients with decreased disc height: loss of disc height, soft tissue buckling, or the presence of foraminal osteophyte of the superior articular process (SAP), endplate lesions such as soft tissue buckling or bony spurs, and instability. Loss of disc height and the presence of endplate lesions contribute to the impingement in the upper to lower direction, and soft tissue buckling or the presence of foraminal osteophytes of SAP and endplate lesions contribute to the impingement in the anterior to posterior direction.

Although many studies have compared the clinical outcomes of indirect decompression between patients with different degrees of spinal stenosis, a consensus regarding its efficacy in severe LSS has not been reached. Therefore, in this retrospective study, we aimed to determine whether the clinical outcomes of indirect decompression through OLIF differed according to the morphological grading of central and foraminal stenosis on preoperative magnetic resonance imaging (MRI) in patients with LSS. The primary outcome was the comparison of clinical scores preoperatively and postoperatively according to the morphological grading of central and foraminal stenosis. In addition, we investigated all factors that can contribute to central or foraminal stenosis, including the above-mentioned factors, and evaluated the risk factors that can lead to clinical dissatisfaction during follow-up after indirect decompression through OLIF, which served as the secondary outcome.

## 2. Materials and Methods

This study was approved by the institutional review board of the Seoul National University Hospital (IRB number: H2207-123-1342), and the need for informed consent was waived by the institutional review board of the Seoul National University Hospital due to the retrospective nature of the study. We retrospectively reviewed the data obtained from consecutive patients with LSS who underwent single-, two-, or three-level OLIF with posterior pedicle screw instrumentation between May 2017 and September 2021. The inclusion criteria were as follows: (1) single-, two-, or three-level OLIF, (2) a minimum of 1 year of follow-up, (3) patients with clinical scores evaluated preoperatively and at 3, 6, and 12 months postoperatively. Patients diagnosed with spondylolytic spondylolisthesis, congenital stenosis, malignancy, inflammatory disease, or infection were excluded. Patients with insufficient clinical data such as the omission of the questionnaire were also excluded from this study.

**Figure 1:**
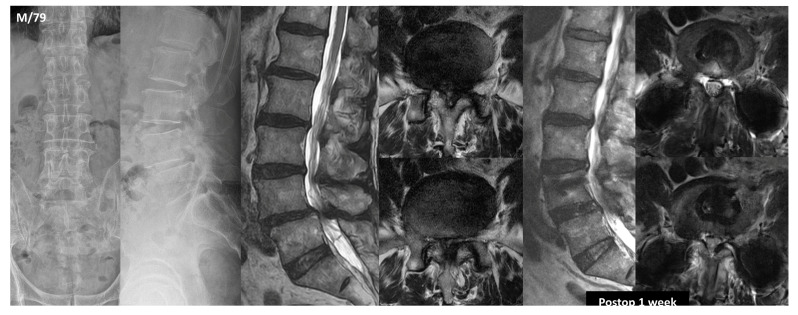
A case of a 79-year-old male with L4–5 spondylolisthesis and severe stenosis demonstrated the efficacy of indirect decompression. This approach effectively improved severe central stenosis, changing Schizas D stenosis to Schizas A or B.

During the study period, OLIF was selected as the primary surgical option for treating LSS when lumbar fusion surgery was required, regardless of the severity of LSS (Figure 1). Direct decompressions, such as posterior lumbar interbody fusion (PLIF) and transforaminal lumbar interbody fusion (TLIF), were considered only in patients who had sequestered IVD causing definite neurologic impairment, a history of previous retroperitoneal surgery, and the presence of blood vessels blocking the L5-S1 IVD in case of L5-S1 level surgery. Patients underwent minimally invasive OLIF using an anterior retroperitoneal pre-psoas approach and received polyether ether ketone cages with demineralized bone matrix, followed by prone-position percutaneous pedicle screw insertion under fluoroscopic guidance or open pedicle screw insertion. In patients with adjacent segment disease (ASD) who had undergone prior fusion, removal of previous fusion metals and posterior instrumentation were executed via the paramedian approach or midline approach. In instances of open posterior instrumentation, direct decompression was not conducted during the index surgery.

Postoperatively, professional rehabilitation was not routinely administered. Instead, patients were encouraged to begin independent walking with the aid of a walker starting from the first day after surgery. Except for activities involving lumbar flexion, no restrictions were placed on daily activities, including walking.

Data on patient-related factors, including demographics and body mass index (BMI), and treatment-related factors, such as the number of surgeries, previous operation history at the index surgical level, posterior fusion surgery, additional posterior decompression surgery, and occurrence of perioperative complications, were obtained from the electronic medical records. As for the clinical scores, the visual analog scale for back pain (VAS-BP), the visual analog scale for leg pain (VAS-LP), the Oswestry Disability Index (ODI) [17], and the Japanese Orthopedic Association Back Pain Evaluation Questionnaire (JOABPEQ) [18] evaluated preoperatively, and 3, 6, 12, 24 months postoperatively, were used for analysis. The proportion of patients attaining the substantial clinical benefit (SCB) framework was assessed based on criteria outlined in Supplementary Table S1 [19,20].

As for the radiological assessment, preoperative axial and sagittal T1- and T2-weighted images were reviewed by two authors who were unaware of the clinical outcomes. We classified the severity of stenosis based on preoperative MRI at the surgical level using qualitative grading systems, such as Schizas’s classification for central stenosis and Lee’s classification for foraminal stenosis (Table 1) [21,22]. In cases of two-level or three-level OLIF, the most severe stenotic lesions in the central canal and neural foramen that could explain the patient’s symptoms were defined as maximal central and maximal foraminal stenosis, respectively. The maximum grades of central and foraminal stenosis were used in the analysis. We also assessed other radiological parameters, including the presence of facet cysts, sequestrated IVDs, and cage subsidence, as well as evaluated the potential causes of central or foraminal stenosis such as soft tissue buckling or the presence of foraminal osteophytes of SAP, endplate lesions of soft tissue buckling such as protruding annulus fibrosus, endplate lesions of bony spurs, instability, and loss of disc height. Instability was defined by a range of motion (ROM) exceeding 10 degrees or vertebral translation greater than 4 mm anteriorly or 2 mm posteriorly. Loss of disc height was quantified as a greater than 30% reduction relative to the adjacent normal disc height.

For statistical analysis, the difference in continuous data among the groups was assessed using Student’s *t*-test, one-way ANOVA with post hoc Bonferroni adjustments, and the Kruskal-Wallis test. Differences in categorical data were assessed using the chi-squared test and linear-by-linear association. Improvements in clinical scores over time were assessed using the paired *t*-test and Wilcoxon signed-rank test. Factors associated with SCB (such as age, sex, BMI, the maximum grade of central and foraminal stenosis, the presence of severe central or foraminal stenosis, the number of surgeries, the co-existence of degenerative spondylolisthesis, previous operation history at the index’s surgical level, posterior fusion surgery, additional posterior decompression surgery, the presence of facet cysts, sequestrated IVDs, cage subsidence, soft tissue buckling, the presence of foraminal osteophytes of SAP, endplate lesions such as soft tissue buckling or bony spurs, instability, loss of disc height, postoperative radiating pain, and postoperative infection) were examined using a logistic regression model. Variables associated with SCB (*p* < 0.20) in the univariate logistic regression analysis were re-entered into the multivariate logistic regression model, which was used to calculate the odds ratios (ORs) and 95% confidence intervals (CIs) of the variables to predict SCB using the backward elimination method. IBM SPSS Statistics version 25.0 (IBM, Armonk, NY, USA) was used for the statistical analysis.

## 3. Results

This study included 145 patients with a mean age ± standard deviation (SD) of 68.93 ± 7.72 years. The mean follow-up was 33.7 ± 13.6 months. Among these, 72 (49.7%) patients underwent single-level OLIF, 49 (33.8%) underwent two-level OLIF, and 24 (16.6%) underwent three-level OLIF. Preoperative MRI revealed that 55 (37.9%) patients had Schizas grade D central stenosis while 74 (51.0%) had Lee grade 3 foraminal stenosis. The patients’ demographic and radiologic characteristics, according to the severity of central and foraminal stenosis on the preoperative MRI, are summarized in Table 2. No significant differences in age, sex, and BMI were observed between the groups stratified by the maximum grade of central stenosis. The follow-up period was significantly higher in patients with grade A maximal central stenosis than in the other groups. Higher grades of maximal central stenosis are significantly associated with increased rates of degenerative spondylolisthesis and instability. Patients with grade B maximal central stenosis showed a significantly higher proportion of patients with facet cysts. No significant variations in age, sex, BMI, or follow-up duration were noted across groups stratified by maximum foraminal stenosis grade. Patients with grade 3 maximal foraminal stenosis showed a significantly higher proportion of patients with a history of previous operations at the index surgical level and significantly higher rates of patients with bony spurs of SAP. The incidence of degenerative spondylolisthesis and instability was significantly higher in patients with grade 0 or 1 maximal foraminal stenosis compared to those with grade 2 or 3. The number of fused levels was significantly higher in patients with grade 2 or 3 maximal foraminal stenosis compared to those with grade 0 or 1.

**Figure 2:**
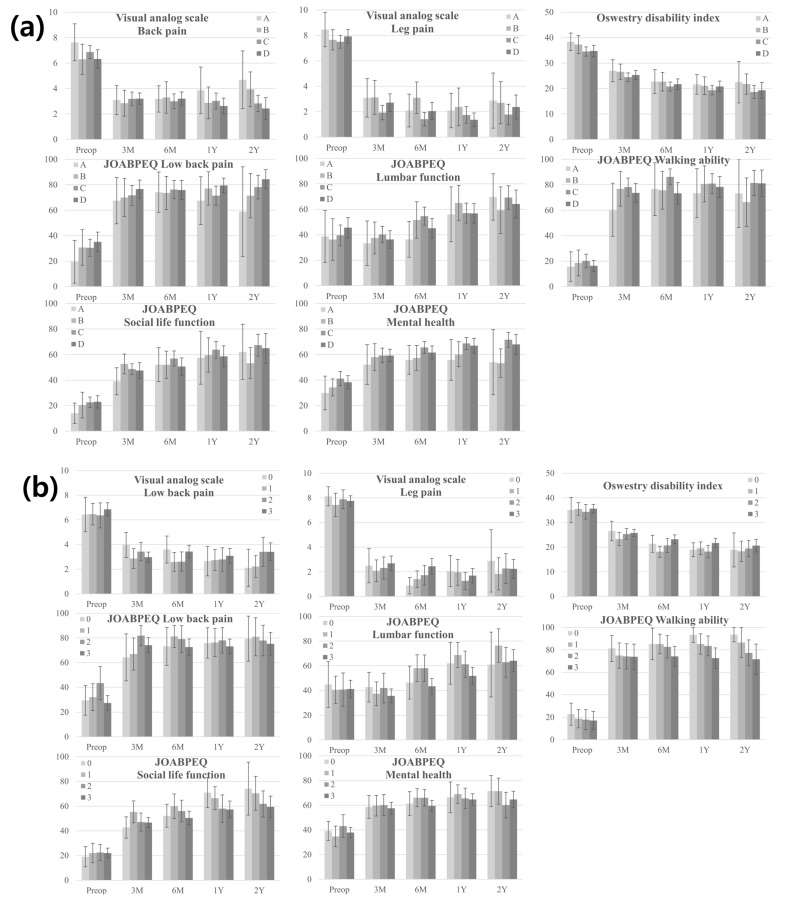
Preoperative and postoperative VAS, ODI, and JOABPEQ scores according to the (**a**) maximum grade of central or (**b**) foraminal stenoses. No significant difference is observed in clinical scores according to the maximum grade of central and foraminal stenoses. Error bars show the range of a 95-percentile confidence interval. VAS, visual analog scale; ODI, Oswestry Disability Index; JOABPEQ, Japanese Orthopedic Association Back Pain Evaluation Questionnaire.

After stratifying the patients based on the maximum grade of central stenosis, all clinical scores showed significant improvement during the 1-year follow-up period except for the lumbar function score in JOABPEQ in patients with grade A maximal central stenosis (*p* = 0.238) (Figure 2a, Supplementary Table S2). No significant difference was observed in clinical scores between postoperative years 1 and 2 (Figure 2a). The most significant improvement was observed in the VAS-LP and walking ability scores in JOABPEQ for all groups. No significant difference was observed in the clinical scores according to the maximum grade of central stenosis at any time point (Supplementary Table S2). Subgroup analysis comparing the groups of patients with severe central stenosis (Schizas grade D central stenosis) with those without severe central stenosis showed that there was no significant difference in the preoperative and postoperative scores between the two groups (Supplementary Table S3).

**Figure 3:**
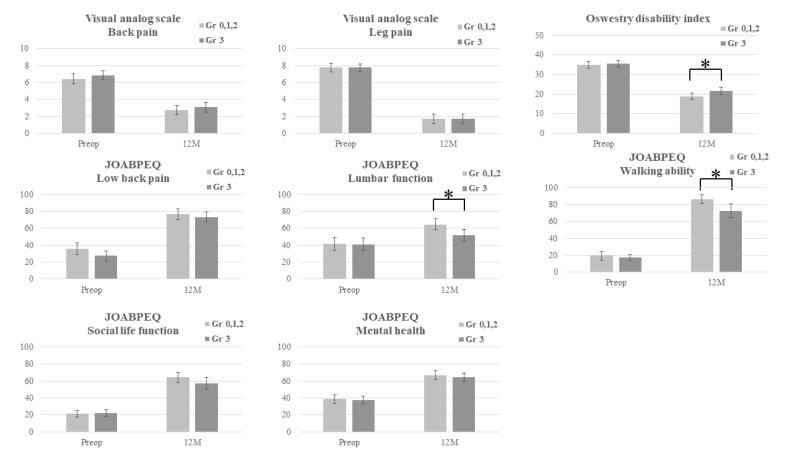
VAS, ODI, and JOABPEQ scores at preoperative and one-year postoperative intervals were stratified by the presence of severe foraminal stenosis. The group with severe foraminal stenosis showed significantly higher ODI (*p* = 0.024) as well as a lower lumbar function score and walking ability score in JOABPEQ (*p* = 0.009 and 0.004, respectively) 1 year postoperatively. * Error bars represent the 95-percentile confidence interval. VAS, visual analog scale; ODI, Oswestry Disability Index; JOABPEQ, Japanese Orthopedic Association Back Pain Evaluation Questionnaire.

When patients were stratified based on the maximum grade of foraminal stenosis, all clinical scores showed significant improvement during the 1-year follow-up period, except for the lumbar function score in JOABPEQ in patients with grade 0 maximal foraminal stenosis (*p* = 0.105) (Figure 2b, Supplementary Table S4). No significant difference was observed in clinical scores between postoperative years 1 and 2 (Figure 2b). No significant difference was observed in the clinical scores according to the maximum grade of foraminal stenosis at any time point among the four groups (Supplementary Table S4). Subgroup analysis comparing the group of patients with severe foraminal stenosis (Lee grade 3 foraminal stenosis) to the other groups showed that the group with severe foraminal stenosis showed a significantly higher ODI score (mean ± SD: 21.7 ± 8.0 versus 18.9 ± 6.4; *p* = 0.024), lower lumbar function score in JOABPEQ (mean ± SD: 51.8 ± 29.6 versus 41.1 ± 30.8; *p* = 0.009), and lower walking ability score in JOABPEQ (mean ± SD: 72.6 ± 34.8 versus 86.4 ± 21.3; *p* = 0.004) at one year postoperatively (Figure 3, Supplementary Table S5).

**Figure 4:**
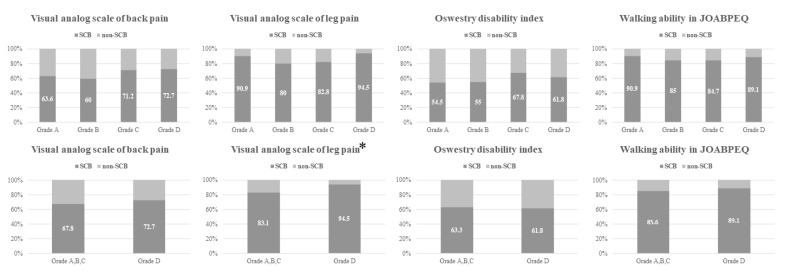
Proportion of patients achieving SCB stratified according to the maximum grade of central stenosis or the existence of severe central stenosis. The number in each column shows the percentage. The group with severe central stenosis showed a significantly higher proportion of patients achieving SCB in VAS-LP. * SCB, substantial clinical benefit; VAS-LP, visual analog scale for leg pain.

**Figure 5:**
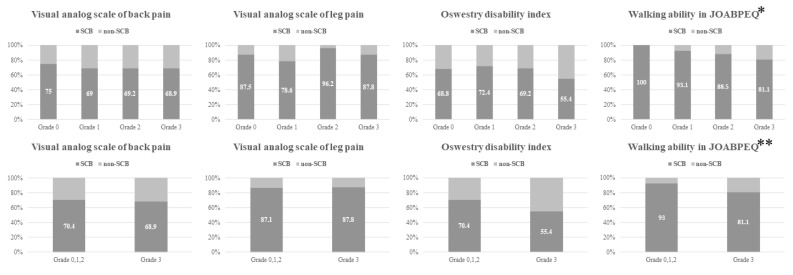
The proportion of patients achieving SCB after stratification by the maximum grade of foraminal stenosis or the existence of severe foraminal stenosis. The number in each column shows the percentage. The proportion of patients achieving SCB in the walking ability score of JOABPEQ showed significant association according to the maximum grade of foraminal stenosis * and the existence of severe foraminal stenosis. ** SCB, substantial clinical benefit; JOABPEQ, Japanese Orthopedic Association Back Pain Evaluation Questionnaire.

The proportions of patients achieving SCB in the VAS-BP, VAS-LP, ODI, and JOABPEQ 1 year postoperatively according to the maximum grade of central and foraminal stenosis are shown in Figure 4 and Figure 5. No significant difference was observed in the proportion of patients achieving SCB according to the maximum grade of central stenosis. When stratified by the presence of severe central stenosis, the group with severe central stenosis showed a significantly higher proportion of patients achieving SCB in VAS-LP (94.5% versus 83.1%; *p* = 0.044) (Figure 4). On the other hand, the proportion of patients achieving SCB in the walking ability score of the JOABPEQ, measured 1 year postoperatively, showed a significant association with the maximum grade of foraminal stenosis (*p* = 0.018) (Figure 5). When stratified by the presence of severe foraminal stenosis, patients with severe foraminal stenosis showed a significantly lower proportion of patients achieving SCB in the walking ability score in the JOABPEQ than those without severe foraminal stenosis (81.1% versus 93.0%; *p* = 0.034) (Figure 5).

Logistic regression analysis was performed to assess achieving SCB in clinically important clinical scores such as the ODI and walking ability score in JOABPEQ, which also showed a significant difference according to the presence of severe foraminal stenosis. In multivariate logistic regression analyses performed for ODI scores, age and the presence of foraminal osteophytes of SAP (OR [95% CI]: 0.92 [0.88–0.98] and 0.20 [0.05–0.81], respectively) were significant risk factors for not achieving SCB in ODI at one year postoperatively (Table 3, full version in Supplementary Table S6). In contrast, degenerative spondylolisthesis (OR [95% CI]: 2.68 [1.23–5.84]) was a significant factor for achieving SCB in the ODI at one year postoperatively. In multivariate logistic regression analyses performed for the walking ability score in JOABPEQ, previous operation history at the index surgical level and the presence of foraminal osteophytes of SAP (OR [95% CI]: 0.15 [0.05–0.45] and 0.22 [0.06–0.86], respectively) were significant risk factors for not achieving SCB in the walking ability score in JOABPEQ at one year postoperatively (Table 4, full version in Supplementary Table S7).

During the follow-up period, 3 cases (2.1%) were diagnosed with postoperative infectious spondylitis; 3 cases (2.1%) presented with pseudo-hernia; 3 cases (2.1%) exhibited sympathetic injury; 4 cases (2.8%) exhibited screw loosening; 6 cases (4.1%) required revision surgery: 2 (1.4%) for ASD, 2 (1.4%) for symptomatic screw loosening, and 2 for postoperative neurological deficits necessitating additional posterior decompression.

## 4. Discussion

In our study, the clinical outcomes of indirect decompression in patients with severe central stenosis were comparable to those in patients without severe central stenosis. Our results showed a significantly higher proportion of patients with severe central stenosis achieving SCB in VAS-LP. These results indicate that the effects of OLIF in patients with severe central stenosis are comparable to those with mild-to-moderate central stenosis. These findings are consistent with those of previous studies showing that severe central stenosis diagnosed by preoperative MRI is not a contraindication for indirect decompression because indirect decompression provides successful clinical results, including restoration of disc height and indirect expansion of the dural sac [15,16]. On the other hand, Oliveira et al. described OLIF as a relative contraindication in severe central stenosis unless the patient accepts the possibility of additional posterior decompression due to incomplete indirect decompression [23]. Li et al. reported that patients with severe central stenosis showed similar radiological decompression effects after indirect decompression as in those without severe central stenosis; however, they needed additional posterior decompression to obtain satisfactory clinical results [10,13]. One possible explanation for these discrepancies is that there could be patient factors that prohibit indirect decompression. Indirect decompression surgery may not be effective when OLIF is performed on patients with maintained disc height because it is difficult to increase the disc height in these patients [24]. Therefore, the appropriate selection of patients for whom indirect decompression can be effective is important.

In our study, patients with severe foraminal stenosis showed significantly higher ODI scores and lower walking ability and lumbar function scores in JOABPEQ 1-year postoperatively. These findings are consistent with those of previous reports (in that indirect decompression may result in poor outcomes in patients with severe foraminal stenosis). Choi et al. showed that 13 of 200 patients who received anterior lumbar interbody fusion had poor clinical outcomes, and 12 of them had residual foraminal stenosis due to incomplete foraminal decompression [25]. Rentenberger et al. reported that patients with foraminal stenosis were more likely to undergo early revision surgery after indirect decompression, primarily because of neurological symptoms or radiating pain [26]. Li et al. also showed that severe foraminal stenosis increases the risk of additional posterior decompression [13]. Although not statistically significant, indirect decompression might have inadequate outcomes in patients with severe foraminal stenosis, considering the lower proportion of those patients achieving SCB in ODI 1 year postoperatively than patients without severe foraminal stenosis (55.4% versus 70.4%, *p* = 0.062).

Buckland et al. reported that patients with foraminal osteophytes extending from the posterior to the cephalad vertebral body showed inferior outcomes following indirect decompression [12]. However, in our experience, the SAP tip forms the posterior margin of the neural foramen in patients with decreased disc height. Multivariate regression analysis showed that the presence of foraminal osteophytes of SAP is a significant risk factor for not achieving SCB in the ODI and the walking ability score in JOABPEQ 1 year postoperatively. This indicates that indirect decompression performed to restore disc height in the superior to inferior direction is not effective in the presence of foraminal osteophytes of SAP that press the nerve root in the anterior-to-posterior direction. Even in patients with co-existing instability, the foraminal osteophytes of SAP can impinge the nerve root after the reduction of instability. Therefore, surgeons should consider direct decompression in the presence of foraminal osteophytes of SAP as well as explain the possibility of additional posterior decompression before indirect decompression in patients with severe foraminal stenosis. In cases where foraminal osteophytes of SAP are absent, placing the cage on the posterior part of the vertebral body during OLIF surgery to increase the height of the intervertebral foramen has been proposed to prevent incomplete foraminal decompression [27]. We recommend confirming the increase in foraminal height after cage insertion on the intraoperative C-arm.

Although previous studies have shown that the effect of indirect decompression decreases when accompanied by spondylolisthesis [11,28], the logistic regression analysis in our study showed that degenerative spondylolisthesis (OR [95% CI]: 2.68 [1.23–5.84]) was a significant factor for achieving SCB in ODI 1 year postoperatively and did not affect the walking ability score in JOABPEQ 1-year postoperatively. This result is consistent with the findings of previous studies that reported that OLIF has shown stronger corrective power than TLIF or PLIF in stabilization and disc height restoration [5,29,30,31]. Severe facet arthropathy and bony lateral recess stenosis cases have been reported, showing a high possibility of poor prognosis [25,32]. Moreover, indirect decompression has also been reported as effective even in the presence of facet degeneration and facet tropism [33].

Only two patients underwent additional posterior decompression. One patient showed inadequate restoration of disc height due to severe osteoporosis and endplate fracture, resulting in no increase in foraminal height on the intraoperative C-arm, and posterior decompression was performed immediately. The other patient showed immediate postoperative radiculopathy due to the insertion of a cage that was placed too posteriorly, which resulted in the narrowing of the contralateral neural foramen. Although not included in this study due to follow-up loss or the omission of the questionnaire, several patients received additional posterior decompressions because of the presence of hook-like foraminal osteophytes of SAP or inferior endplates, which caused new root irritation as the disc height increased, or because of cauda equina syndrome, caused by a postoperative herniated disc after cage placement with insufficient posterior discectomy.

This study has several limitations. First, since it is a retrospective study and patients with spondylolytic spondylolisthesis were excluded, this may have impacted the results. However, because patients with spondylolytic spondylolisthesis generally exhibit foraminal stenosis without central stenosis, and their anatomical structures respond differently to the restoration of disc height in indirect decompression, owing to lysis of the pars interarticularis, we believe that the outcomes of OLIF in patients with severe central and foraminal stenoses could be evaluated more accurately by excluding them. Second, radiologic evaluation, including sagittal alignment before and after surgery, was not performed. Sagittal imbalance is closely related to the occurrence of ASD; therefore, the influence of the sagittal profile was not considered. However, OLIF itself is more effective for correcting sagittal imbalance than other fusion surgeries, such as PLIF or TLIF [34]. Only 2 cases (1.4%) of ASD underwent revision surgery during the follow-up period in our study. Therefore, this omission is not expected to have a significant impact on the conclusion of our study. Third, the clinical scores at 2 years postoperatively were omitted in 47 patients (32.4%). However, the 1-year postoperative clinical scores showed no significant difference from the 2-year postoperative clinical scores. Moreover, given the persuasiveness and power of regression analysis, utilizing one-year clinical scores is advantageous due to the greater data volume, justifying the use of one-year data for robust regression analysis. Finally, the lack of data on the physical activity levels after OLIF and the absence of a professional rehabilitation protocol postoperatively limit our understanding of these factors as potential confounders. Future studies should include standardized rehabilitation programs to better assess their impacts on recovery and long-term outcomes.

## 5. Conclusions

In the current study, patients with severe central stenosis showed clinical outcomes comparable to those with mild-to-moderate central stenosis after OLIF. However, the improvement in the ODI and the walking ability score in JOABPEQ after OLIF was limited in patients with severe foraminal stenosis. Surgeons should consider direct decompression if the foraminal osteophytes of SAP are present. Additionally, they should explain the possibility of additional posterior decompression before indirect decompression in patients with severe foraminal stenosis.

## Figures and Tables

**Table 1:** The qualitative grading systems of lumbar spinal stenosis on magnetic resonance imaging.

	Grade A	Grade B	Grade C	Grade D
Central lesion [21]	Mild stenosis and visible CSF	Moderate stenosis and visible rootlets	Severe stenosis and invisible rootlets	Extreme stenosis, invisible rootlets, and no epidural fat
	**Grade 0**	**Grade 1**	**Grade 2**	**Grade 3**
Foraminal lesion [22]	Normal	Perineural fat obliteration in the two opposing directions	Perineural fat obliteration in the four directions	Nerve root collapse or morphologic changes

CSF, cerebrospinal fluid.

**Table 2:** Demographic characteristics according to stenosis grade.

Index	Maximum Grade of Central Stenosis				
	A (n = 11)	B (n=20)	C (n = 59)	D (n = 55)	*p*-Value
Age (mean ± SD, years)	66.27 ± 9.96	69.15 ± 6.92	67.53 ± 8.09	70.89 ± 6.77	0.076
Sex, M:F (n)	3:8	4:16	22:37	16:39	0.496
BMI (mean ± SD, kg/m^2^)	24.88 ± 7.39	22.23 ± 4.96	25.83 ± 6.74	24.74 ± 6.37	0.201
**Indexes**	**Maximum Grade of Foraminal Stenosis**				
	**0 (n = 16)**	**1 (n = 29)**	**2 (n = 26)**	**3 (n = 74)**	***p*-Value**
Age (mean ± SD, years)	67.06 ± 7.8	66.59 ± 9.42	70.5 ± 7.39	69.7 ± 6.91	0.142
Sex, M:F (n)	4:12	7:22	11:15	23:51	0.482
BMI (mean ± SD, kg/m^2^)	23.33 ± 7.8	25.97 ± 6.76	25.54 ± 7.44	24.49 ± 5.69	0.522

SD, standard deviation; M:F, male:female.

**Table 3:** Multivariate logistic regression analyses were performed to assess the achievement of substantial clinical benefit (SCB) in the Oswestry Disability Index (ODI) 1 year postoperatively.

Characteristics	OR (95% CI)	*p*-Value
Age (years)	**0.92 (0.88–0.98)**	**0.004**
Preoperative diagnosis		**0.040**
Degenerative spondylolisthesis *	**2.68 (1.23–5.84)**	**0.013**
Adjacent segment disease *	1.15 (0.25–5.40)	0.856
Previous operation history in index surgical level		0.240
Open posterior fusion		0.124
The number of surgical levels		0.307
Severe foraminal stenosis		0.547
Foraminal osteophytes of SAP	**0.20 (0.05–0.81)**	**0.024**
Instability		0.420

* Odds compared to spinal stenosis without spondylolisthesis; OR, odds ratio; CI, confidence interval.

**Table 4:** Multivariate logistic regression analyses were performed to assess the achievement of substantial clinical benefit (SCB) in the walking ability score in the Japanese Orthopedic Association Back Pain Evaluation Questionnaire (JOABPEQ) 1 year postoperatively.

Characteristics	OR (95% CI)	*p*-Value
Previous operation history in index surgical level	**0.15 (0.05–0.45)**	**0.001**
Open posterior fusion		0.344
Maximum grade of foraminal stenosis		0.478
Severe foraminal stenosis		0.415
Foraminal osteophytes of SAP	**0.22 (0.06–0.86)**	**0.030**
Facet cyst		0.115
Cage subsidence		0.191

OR, odds ratio; CI, confidence interval; SAP, superior articular process.

## Data Availability

Data underlying this article cannot be shared publicly due to the privacy of the individuals who participated in this study. The data may be shared upon reasonable request to the corresponding author.

## Supplementary Materials

The following supporting information can be downloaded at: https://www.mdpi.com/article/10.3390/jcm13154421/s1. Supplementary Table S1. Criteria for substantial clinical benefit (SCB) in the clinical scores. Supplementary Table S2. Preoperative and one-year postoperative clinical scores stratified by the maximum grade of central stenosis. Supplementary Table S3. Preoperative and one-year postoperative clinical scores stratified by the existence of severe central stenosis. Supplementary Table S4. Preoperative and one-year postoperative clinical scores stratified by the maximum grade of foraminal stenosis. Supplementary Table S5. Preoperative and one-year postoperative clinical scores stratified by the existence of severe foraminal stenosis. Supplementary Table S6. Univariate and multivariate logistic regression analyses on achieving substantial clinical benefit (SCB) in Oswestry disability index (ODI) 1 year postoperatively. Supplementary Table S7. Univariate and multivariate logistic regression analyses on achieving substantial clinical benefit (SCB) in the walking ability score in JOABPEQ 1 year postoperatively.
