# Supplementary material for: Effects of the Severity of Stenosis on Clinical Outcomes of Indirect Decompression Using Oblique Lumbar Interbody Fusion
Source: J Clin Med. 2024 Jul 28;13(15):4421. doi: 10.3390/jcm13154421 (PMC11313687; doi:10.3390/jcm13154421)
Supplement: Supplementary file 1 [file jcm-13-04421-s001.zip › jcm-3091879-supplementary.pdf]

Supplementary Table S1. Criteria for substantial clinical benefit (SCB) in the clinical scores

Clinical score	Criteria for substantial clinical benefit (SCB) in the clinical score
VAS-BP	2.5-point net improvement or 41.4% percent improvement (Glassman et al.; 2008)
VAS-LP	2.5-point net improvement or 38.8% percent improvement (Glassman et al.; 2008)
ODI	18.8-point net improvement or 36.8% percent improvement (Glassman et al.; 2008)
JOABPEQ	20-point net improvement (Kasai et al.; 2017)

VAS-BP, visual analog scale for back pain; VAS-LP, visual analog scale for leg pain; ODI, Oswestry disability index; JOABPEQ, Japanese Orthopedic Association Back Pain Evaluation Questionnaire

Supplementary Table S2. Preoperative and one-year postoperative clinical scores stratified by the maximum grade of central stenosis

Clinical scores		The maximum grade of central stenosis							
		A (n=11)		B (n=20)		C (n=59)		D (n=55)	
		Preoperative	Postoperative 1-year	Preoperative	Postoperative 1-year	Preoperative	Postoperative 1-year	Preoperative	Postoperative 1-year
ODI		38.4 ± 5.1	21.6 ± 5.7	37.3 ± 7.7	21.1 ± 7.4	34.6 ± 7.0	19.3 ± 7.0	34.8 ± 8.1	20.8 ± 7.8
VAS	BP	7.6 ± 2.2	3.9 ± 2.7	6.3 ± 2.5	2.9 ± 2.7	6.9 ± 2.0	3.0 ± 2.4	6.3 ± 2.7	2.6 ± 2.3
	LP	8.5 ± 2.0	2.1 ± 2.0	7.7 ± 1.7	2.4 ± 3.2	7.5 ± 2.0[a]	1.7 ± 2.5[a]	7.9 ± 2.0[a]	1.4 ± 2.0[a]
JOABPEQ	LBP	19.5 ± 25.0	67.5 ± 27.9	30.7 ± 29.8	77.1 ± 28.0	30.5 ± 25.7	71.4 ± 28.8	35.1 ± 28.8	79.5 ± 21.3
	LF	38.6 ± 30.3[b]	56.1 ± 32.1[b]	36.3 ± 35.0	65.0 ± 29.8	39.6 ± 31.4	57.1 ± 30.0	45.6 ± 29.7	56.8 ± 28.2
	WA	15.6 ± 17.4	73.4 ± 28.8	15.6 ± 21.7	80.7 ± 30.2	20.2 ± 20.2[a]	81.0 ± 29.9[a]	16.4 ± 15.7[a]	78.3 ± 30.0[a]
	SLF	14.0 ± 11.9	57.5 ± 30.8	14.0 ± 21.6	59.6 ± 28.7	22.6 ± 16.1[a]	63.7 ± 25.2[a]	23.0 ± 17.8	58.7 ± 29.8
	MH	29.9 ± 19.6	55.8 ± 23.8	29.9 ± 14.2	60.3 ± 20.9	41.2 ± 21.3	68.6 ± 18.1	38.4 ± 19.1	66.9 ± 21.4

All scores are presented as mean ± standard deviation.; All preoperative scores showed significant improvement at 1-year postoperatively for paired *t*-tests or Wilcoxon signed rank tests[a] ($P < 0.05$) except JOABPEQ_LF in grade 0 of maximal central stenosis ($P = 0.238$)[b]. No significant difference in preoperative and postoperative scores for one-way ANOVA test or Kruskal-Wallis test is observed between four groups.

ODI, Oswestry disability index; VAS-BP, visual analog scale for back pain; VAS-LP, visual analog scale for leg pain; JOABPEQ, Japanese Orthopedic Association Back Pain Evaluation Questionnaire; LBP, lower back pain; LF, lumbar function; WA, walking ability; SLF, social life function; MH, mental health

Supplementary Table S3. Preoperative and one-year postoperative clinical scores stratified by the existence of severe central stenosis

		The maximum grade of central stenosis			
		A, B, or C (n = 90)		D (n = 55)	
Clinical scores		Preoperative	Postoperative 1-year	Preoperative	Postoperative 1-year
ODI		35.6 ± 7.0	20.0 ± 7.0	34.8 ± 8.1	20.8 ± 7.8
VAS	BP	6.8 ± 2.2	3.1 ± 2.5	6.3 ± 2.7	2.6 ± 2.3
	LP	7.7 ± 1.9[a]	1.9 ± 2.6[a]	7.9 ± 2.0[a]	1.4 ± 2.0[a]
JOABPEQ	LBP	29.2 ± 26.5[a]	72.2 ± 28.4[a]	35.1 ± 28.8	79.5 ± 21.3
	LF	38.7 ± 31.7	58.7 ± 30.1	45.6 ± 29.7	56.8 ± 28.2
	WA	19.3 ± 20.1[a]	80 ± 29.6[a]	16.4 ± 15.7[a]	78.3 ± 30.0[a]
	SLF	21.1 ± 17.1	62.0 ± 26.5	23.0 ± 17.8	58.7 ± 29.8
	MH	38.3 ± 20.0	65.2 ± 19.9	38.4 ± 19.1	66.9 ± 21.4

All scores are presented as mean ± standard deviation. All preoperative scores showed significant improvement at 1-year postoperatively for paired *t*-tests or Wilcoxon signed rank tests[a] ($P < 0.05$). There is no significant difference in preoperative and postoperative scores for student *t*-test between two groups.

ODI, Oswestry disability index; VAS-BP, visual analog scale for back pain; VAS-LP, visual analog scale for leg pain; JOABPEQ, Japanese Orthopedic Association Back Pain Evaluation Questionnaire; LBP, lower back pain; LF, lumbar function; WA, walking ability; SLF, social life function; MH, mental health

Supplementary Table S4. Preoperative and one-year postoperative clinical scores stratified by the maximum grade of foraminal stenosis

		The maximum grade of foraminal stenosis							
		0 (n=16)		1 (n=29)		2 (n=26)		3 (n=74)	
Clinical scores		Preoperative	Postoperative 1-year	Preoperative	Postoperative 1-year	Preoperative	Postoperative 1-year	Preoperative	Postoperative 1-year
ODI		35.1 ± 9.6	19.0 ± 5.3	35.5 ± 6.8	19.5 ± 7.0	34.4 ± 7.3	18.2 ± 6.3	35.6 ± 7.3	21.7 ± 8.0
VAS	LBP	6.4 ± 2.6	2.7 ± 2.3	6.5 ± 2.3	2.7 ± 2.3	6.4 ± 2.5	2.8 ± 2.4	6.9 ± 2.3	3.1 ± 2.6
	LP	8.1 ± 1.5	2.1 ± 2.4	7.4 ± 2.5[a]	2.0 ± 2.8[a]	7.9 ± 1.9	1.3 ± 1.7	7.8 ± 1.8[a]	1.7 ± 2.5[a]
JOABPEQ	LBP	29.5 ± 22.4[a]	75.9 ± 23.2[a]	32.0 ± 28.5	76.4 ± 30.1	43.4 ± 33.0	78.0 ± 25.6	27.4 ± 25.1	73.2 ± 25.5
	LF	44.8 ± 34.8[b]	62.0 ± 31.8[b]	40.5 ± 28.9	68.7 ± 26.8	40.7 ± 33.3	61.2 ± 26.6	41.1 ± 30.8[a]	51.8 ± 29.6[a,]
	WA	22.8 ± 18.5	93.3 ± 12.9	18.7 ± 21.5[a]	85.2 ± 23.9[a]	17.9 ± 22.1[a]	83.5 ± 22.0[a]	17.1 ± 16.1[a]	72.6 ± 34.8[a]
	SLF	19.1 ± 15.4	71.0 ± 22.9	22.0 ± 20.8	66.5 ± 24.6	22.5 ± 15.9	58.0 ± 27.4	22.0 ± 17.1[a]	57.3 ± 29.5[a]
	MH	39.2 ± 14.6	66.4 ± 23.3	34.7 ±22.0	69.0 ± 19.7	43.1 ± 22.8	65.5 ± 20.6	37.8 ± 18.4	64.6 ± 20.2

All scores are presented as mean ± standard deviation. All preoperative scores showed significant improvement at 1-year postoperatively for paired *t*-tests or Wilcoxon signed rank tests[a] ($P < 0.05$) except JOABPEQ_LF in grade 0 of maximal foraminal stenosis ($P = 0.105$)[b]. There is no significant difference in preoperative and postoperative scores for a one-way ANOVA test or Kruskal-Wallis test between four groups.

ODI, Oswestry disability index; VAS-BP, visual analog scale for back pain; VAS-LP, visual analog scale for leg pain; JOABPEQ, Japanese Orthopedic Association Back Pain Evaluation Questionnaire; LBP, lower back pain; LF, lumbar function; WA, walking ability; SLF, social life function; MH, mental health

Supplementary Table S5. Preoperative and one-year postoperative clinical scores stratified by the existence of severe foraminal stenosis

		The maximum grade of foraminal stenosis			
		0, 1, or 2 (n = 71)		3 (n = 74)	
Clinical scores		Preoperative	Postoperative 1-year	Preoperative	Postoperative 1-year
ODI		35.0 ± 7.6	18.9 ± 6.3[b]	35.6 ± 7.3	21.7 ± 8.0[b]
VAS	BP	6.4 ± 2.4	2.7 ± 2.3	6.8 ± 2.3	3.1 ± 2.6
	LP	7.8 ± 2.1[a]	1.7 ± 2.3[a]	7.8 ± 1.8[a]	1.7 ± 2.5[a]
JOABPEQ	LBP	35.6 ± 29.3[a]	76.9 ± 26.7[a]	27.4 ± 25.1	73.2 ± 25.5
	LF	41.6 ± 31.5	64.4 ± 27.7[b]	41.1 ± 30.8[a]	51.8 ± 29.6[a,b]
	WA	19.3 ± 20.9[a]	86.4 ± 21.3[a,b]	17.1 ± 16.1[a]	72.6 ± 34.8[a,b]
	SLF	21.5 ± 17.8	64.4 ± 25.5	22.0 ± 17.1[a]	57.3 ± 29.5[a]
	MH	38.8 ± 20.9	67.1 ± 20.6	37.8 ± 18.4	64.6 ± 20.2

All scores are presented as mean ± standard deviation. All preoperative scores showed significant improvement at 1-year postoperatively for paired *t*-tests or Wilcoxon signed rank tests[a] ($P < 0.05$). Group with severe foraminal stenosis showed a significantly higher ODI (P=0.024), lesser lumbar function score, and walking ability score in JOABPEQ (P=0.009 and 0.004, respectively) at 1-year postoperative for student *t*-test.[b]

ODI, Oswestry disability index; VAS-BP, visual analog scale for back pain; VAS-LP, visual analog scale for leg pain; JOABPEQ, Japanese Orthopedic Association Back Pain Evaluation Questionnaire; LBP, lower back pain; LF, lumbar function; WA, walking ability; SLF, social life function; MH, mental health

Supplementary Table S6. Univariate and multivariate logistic regression analyses on achieving substantial clinical benefit (SCB) in Oswestry disability index (ODI) 1 year postoperatively.

	Univariate analysis		Multivariate analysis	
	Odds ratio (95% CI)	*P* value	Odds ratio (95% CI)	*P* value
Age (years)	0.92 (0.87–0.97)	**0.001**	**0.92 (0.88-0.98)**	**0.004**
Sex (male)	0.84 (0.41–1.74)	0.645		
BMI	1.00 (0.95–1.05)	0.976		
Preoperative diagnosis		**0.007**		**0.040**
Degenerative spondylolisthesis[a]	3.22 (1.55–6.69)	**0.002**	**2.68 (1.23-5.84)**	**0.013**
Adjacent segment disease[a]	1.47 (0.35–6.12)	0.599	1.15 (0.25-5.40)	0.856
Previous operation history in index surgical level	0.48 (0.20–1.18)	0.111		0.240
Open posterior fusion	0.26 (0.08–0.93)	**0.037**		0.124
The number of surgical level		0.120		0.307
2[b]	0.70 (0.32–1.49)	0.349		
3[b]	0.37 (0.14–0.96)	0.041		
Maximum grade of central stenosis		0.686		
Grade B[c]	1.02 (0.23–4.47)	0.981		
Grade C[c]	1.75 (0.48–6.48)	0.399		
Grade D[c]	1.35 (0.37–4.98)	0.653		
Severe central stenosis	0.94 (0.47–1.87)	0.855		
Maximum grade of foraminal stenosis		0.316		
Grade 1[c]	1.19 (0.31–4.53)	0.795		
Grade 2[c]	1.02 (0.27–3.93)	0.974		
Grade 3[c]	0.57 (0.18–1.79)	0.331		
Severe foraminal stenosis	0.52 (0.26–1.04)	0.063		0.547
Foraminal osteophyte of SAP	0.15 (0.04–0.57)	**0.006**	**0.20 (0.05-0.81)**	**0.024**
Endplate lesion		0.453		
Soft tissue buckling	1.50 (0.72–3.11)	0.281		
Bony spur	0.76 (0.14–4.19)	0.753		
Instability	3.37 (1.66–6.82)	**0.001**		0.420
Decreased disc height		0.257		
Only in standing	5.89 (0.68–50.75)	0.107		
Always	1.02 (0.49–2.13)	0.964		
Facet cyst	0.78 (0.17–3.63)	0.753		
Sequestrated disc	0.62 (0.23–1.63)	0.330		

Cage subsidence	0.92 (0.44–1.90)	0.819		
Postoperative radiating pain	0.88 (0.24–3.28)	0.852		
Postoperative infection	0.89 (0.14–5.48)	0.897		
[a] Odds compared to spinal stenosis without spondylolisthesis, [b] Odds compared to single level, [c] Odds compared to grade A or 0				

ODI, Oswestry disability index; BMI, body mass index; CI, confidence interval; SAP, superior articular plate

Supplementary Table S7. Univariate and multivariate logistic regression analyses on achieving substantial clinical benefit (SCB) in the walking ability score in JOABPEQ 1 year postoperatively.

	Univariate analysis		Multivariate analysis	
	Odds ratio (95% CI)	*P* value	Odds ratio (95% CI)	*P* value
Age (years)	0.96 (0.90–1.03)	0.211		
Sex (male)	1.30 (0.44–3.87)	0.634		
BMI	1.02 (0.94–1.120)	0.678		
Preoperative diagnosis		0.216		
Degenerative spondylolisthesis[a]	1.04 (0.35–3.05)	0.948		
Adjacent segment disease[a]	0.27 (0.05–1.39)	0.118		
Previous operation history in index surgical level	0.14 (0.05–0.40)	**<0.001**	**0.15 (0.05-0.45)**	**0.001**
Open posterior fusion	0.17 (0.05–0.59)	**0.006**		0.344
The number of surgical level		0.754		
2[b]	0.97 (0.34–2.74)	0.951		
3[b]	1.77 (0.36–8.74)	0.481		
Maximum grade of central stenosis		0.877		
Grade B[c]	0.57 (0.05–6.21)	0.642		
Grade C[c]	0.56 (0.06–4.89)	0.596		
Grade D[c]	0.82 (0.09–7.55)	0.858		
Severe central stenosis	1.38 (0.49–3.87)	0.542		
Maximum grade of foraminal stenosis		0.130		0.478
Grade 1[c]		0.268		
Grade 2[c]		0.794		
Grade 3[c]		**0.034**		
Severe foraminal stenosis	0.33 (0.11–0.96)	**0.041**		0.415
Foraminal osteophyte of SAP	0.19 (0.06–0.66)	**0.009**	**0.22 (0.06-0.86)**	**0.030**
Endplate lesion		0.355		
Soft tissue buckling	0.39 (0.11–1.43)	0.155		
Bony spur	0.37 (0.03–4.22)	0.420		
Instability	1.46 (0.56–3.86)	0.442		
Decreased disc height		0.354		
Only in standing	0.30 (0.04–2.10)	0.225		
Always	0.42 (0.11–1.54)	0.190		
Facet cyst	0.18 (0.04–0.85)	**0.031**		0.115
Sequestrated disc	0.78 (0.20–2.96)	0.710		

Cage subsidence	0.43 (0.16–1.14)	0.090		0.191
Postoperative radiating pain	0.58 (0.11–2.94)	0.508		
Postoperative infection	0.59 (0.06–5.58)	0.645		
[a] Odds compared to spinal stenosis without spondylolisthesis, [b] Odds compared to single level, [c] Odds compared to grade A or 0				

ODI, Oswestry disability index; BMI, body mass index; CI, confidence interval; SAP, superior articular plate
